# The first 8 weeks of the Austrian SARS-CoV-2 epidemic

**DOI:** 10.1007/s00508-020-01804-9

**Published:** 2021-02-01

**Authors:** Anna Nagel, Agata Łaszewska, Gerald Haidinger, Judit Simon

**Affiliations:** 1 Department of Health Economics, Center for Public Health, Medical University of Vienna, Kinderspitalgasse 15, 1090 Vienna, Austria; 2 Department of Epidemiology, Center for Public Health, Medical University of Vienna, Vienna, Austria

**Keywords:** COVID-19, Public health measures, Austria, Relative risk, Mental health

## Abstract

**Background:**

Severe acute respiratory syndrome coronavirus 2 (SARS-CoV‑2) reached Austria in February 2020. This study aims to describe the first 8 weeks of the Austrian epidemic and reflect on the potential mental health consequences as known at that time.

**Methods:**

Data on Austrian Coronavirus Disease 19 (COVID-19) epidemiological indicators and number of tests were obtained from official registers. Relative risks (RRs) of infection and death from COVID-19 were calculated for sex and age groups (< 65 years and ≥ 65 years). Public health measures introduced to reduce the spread of COVID-19 were identified via online media research. A rapid review of initial evidence on mental health consequences of the pandemic was performed in PubMed and medRxiv.

**Results:**

By 21 April 2020 the case count in Austria was 14,810 after a peak of new daily infections mid-March. The RR of death for age ≥ 65 years was 80.07 (95% confidence interval, CI 52.64–121.80; *p* < 0.0001) compared to those aged < 65 years. In men the RR of death was 1.44 (95% CI 1.20–1.73; *p* < 0.0001) compared to women. Wide-ranging public health measures included avoidance of case importation, limitation of social contacts, hygiene measures, testing, case tracking, and the call for COVID-19-related research. International rates of psychiatric symptoms during the initial lockdowns exceeded typical levels: anxiety (6%–51%), depression (17%–48%) and posttraumatic stress (5%–54%).

**Conclusion:**

Data show great vulnerability of older people also in Austria. Severe mental health impacts can be expected with need for proper assessment of the long-term consequences of this pandemic.

## Introduction

In December 2019 a series of patients with pneumonia of unknown origin with exposure to the Huanan seafood market in Wuhan was linked to a new coronavirus [1], now known as severe acute respiratory syndrome coronavirus 2 (SARS-CoV-2). The new virus spread internationally within 1 month [2], reached Europe and had formed two hot-spot regions in Northern Italy by the end of February 2020 [3]. Finally, the first two cases in Austria were reported on 25 February [4].

The fast spread of the disease and its deleterious effects on the Italian healthcare system [5] served as an immediate reminder of the urgency of the situation in Austria and underlined the need for measures to prevent an overload of its own healthcare system. Modelling systems were used to predict the beneficial impact of reducing social contacts and thereby decreasing transmission on the epidemic peak [6]. In the hope of achieving this, many nonpharmaceutical public health strategies were combined ranging from minimizing transmission in a personal and professional context to avoiding further importation of new cases [7, 8].

The history of public health response to infectious diseases, in particular that of quarantine, “in the sense of restraining the movement of persons or goods on land or sea due to contagious disease” [9], dates back more than half a millennium. It is thus not a new idea. In the Plague epidemic in the fourteenth century entering certain cities was prohibited and infected individuals were separated from the public. Without knowledge of neither origins nor incubation time of the disease, the common time for isolation was set at 40 days. Similar measures were taken in an attempt to limit the spread of smallpox, yellow fever and cholera. During the Spanish Flu in 1918–1919 the range of disease containment strategies was extended to closure of schools, theatres, churches and suspension of public gatherings. In the severe acute respiratory syndrome (SARS) epidemic in 2003 measures ranged greatly from voluntary quarantine of suspect cases in Canada to cordoning off buildings, installing cameras in private homes and severe sanctions in cases of disobedience in China [9]. Time has advanced as has technology and research. Another chapter in history has been opened with the Coronavirus Disease 2019 (COVID-19) and we will shed light on Austria’s initial 2020 response to this pandemic in the following article.

The severity of the disease, despite extensive international research, remains unclear as it is difficult to assess this in an ongoing pandemic. It seems that older people (aged > 60 years) are more at risk of severe disease and death [10]. Estimates of case fatality ratio, the ratio of deaths to confirmed cases, vary greatly in literature. In one study an adjusted case fatality ratio was estimated at 1.38% [11]. Much higher numbers were estimated for higher age groups: 6.4% among ≥ 60-year-olds up to 13.4% in patients ≥ 80 years of age. Infection fatality ratio, the ratio of deaths to total infections (including asymptomatic infections), was estimated at 0.66%, and it increased with age [11]. Interestingly, the sex ratio in confirmed cases seems to be slightly tilted towards men (1.03:1) [12] and the case fatality ratio might be higher among males as well [13]. It will be examined in the following study if this stays true for the Austrian population.

Beside its impact on mortality, it has been established in extensive disaster mental health research that emotional distress is omnipresent as one of the other main public health impacts in populations affected by a pandemic [14]. To give an example, 48% and 76% of the general public, respectively, had anxiety-depression and posttraumatic stress disorder (PTSD) symptoms after more than 1 year of the Ebola outbreak in Sierra Leone [15]. Several factors such as uncertain predictions, shortages in healthcare supplies, imposition of public health measures severely limiting personal freedom and economic aspects will certainly add to widespread emotional distress and increased risk of psychiatric illness in this pandemic as well. Some groups may be more vulnerable to the deleterious effects on mental health of pandemics than others [14].

This paper, therefore, gives an overview of the first 8 weeks of the Austrian epidemic by reflecting on the main epidemiological indicators including vulnerability of different sex and age groups, the implemented public health measures as well as the initial international evidence on the potential mental health impact of the pandemic and aims to serve as reference paper for these questions.

## Methods

### Evidence on epidemiological indicators

The observational period included time from the appearance of the first cases on 25 February 2020 until 20 April 2020 (8 weeks later) in Austria. Data extraction of the total case counts as well as sex and age distribution were performed on 21 April 2020 from the dashboard of the Federal Ministry of Social Affairs, Health, Care and Consumer Protection (BMSGPK) which provided up to date information about the Austrian SARS-CoV‑2 epidemic [4].

The ministry’s dashboard provided information about latest case counts in the Austrian provinces (*Bundesländer*) and number of performed tests but did not make a timeline accessible for these data. Instead, a summary table of this up to date information retrieved on a daily basis from the BMSGPK’s homepage was available on Wikipedia. These data were used in the following analysis of province-level case counts and number of daily tests [16]. In order to be able to compare the rate of infection between different provinces, the absolute case counts were transformed into numbers per 100,000 inhabitants.

Demographics such as total number of Austrian inhabitants, inhabitants of each of the nine Austrian provinces, as well as sex and age distribution of the Austrian population as of January 2020 were retrieved from Statistics Austria [17]. Relative risks (RR) of infection or death within the population and among cases between different sex and age groups and their confidence intervals were calculated. A case fatality ratio of Austrian cases was calculated by dividing the number of reported deaths by the number of confirmed cases [18]. Calculations of the effective reproduction numbers were downloaded from the homepage of the Austrian Agency for Health and Food Safety (AGES) [19].

### Evidence on implemented public health measures

Information was drawn from online newspaper articles and online government documents.

### Evidence on the mental health impact of the pandemic

Within the process of an initial scoping of the literature about mental health issues during the pandemic, it became evident that three areas were predominantly impacted: anxiety, depression and PTSD/posttraumatic stress symptoms (PTSS). Consequently, the rapid review presented in this paper was restricted to studies reporting on these three disease areas.

The literature search was performed in April in two databases, PubMed and (due to the fast evolving situation) the pre-print server medRxiv using the terms “COVID-19/SARS-CoV-2” + “mental health”, “COVID-19/SARS-CoV-2” + “anxiety”, “COVID-19/SARS-CoV-2” + “depression” and “COVID-19/SARS-CoV-2” + “PTSD”/“PTSS”. In order to be included in the analysis, studies had to be original survey studies published on one of the platforms between 25 February and 20 April 2020 and be using established mental health questionnaires in adult and adolescent general populations to identify the initial mental health effects of COVID-19 and any consequently implemented public health measures. Reviews and interventional studies were excluded as well as articles in other languages than English or German and those for which no abstract was available. Studies were also excluded when the group of interest was mainly healthcare workers, patients with COVID-19 or children. To allow for comparability, studies using not yet validated questionnaires were excluded.

## Results

### Evidence on epidemiological indicators

**Fig. 1:**
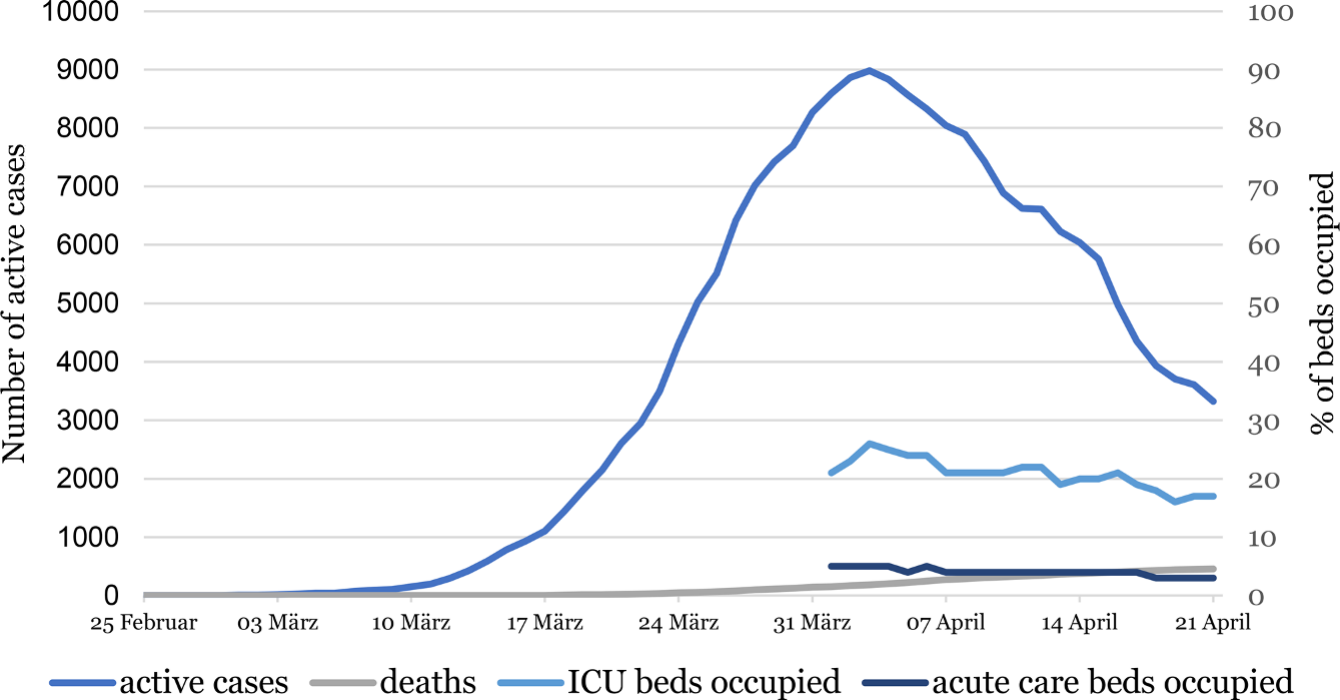
Active COVID-19 cases, deaths and hospital bed occupancy in Austria

The number of active cases decreased after its peak (8981 active cases) in the beginning of April 2020, while the cumulative number of deaths rose to 455 by 20 April 2020 (Fig. 1). Even though the number of active cases declined substantially by 5356 (65%) by 20 April, compared to the peak of active cases, the occupancy of intensive care unit and normal acute care hospital beds (data available only from beginning of April) decreased slower (9 beds less occupied translating to 35% decrease in hospital bed occupancy). This graphically depicts the long aftermath of a surge in infection numbers in hospital bed occupancy. With a cumulative number of 455 deaths and 14,810 persons having been tested positive for SARS-CoV-2 at the point of measurement, the case fatality ratio over the first 8 weeks of the epidemic in Austria was calculated at 3.07%.

**Fig. 2:**
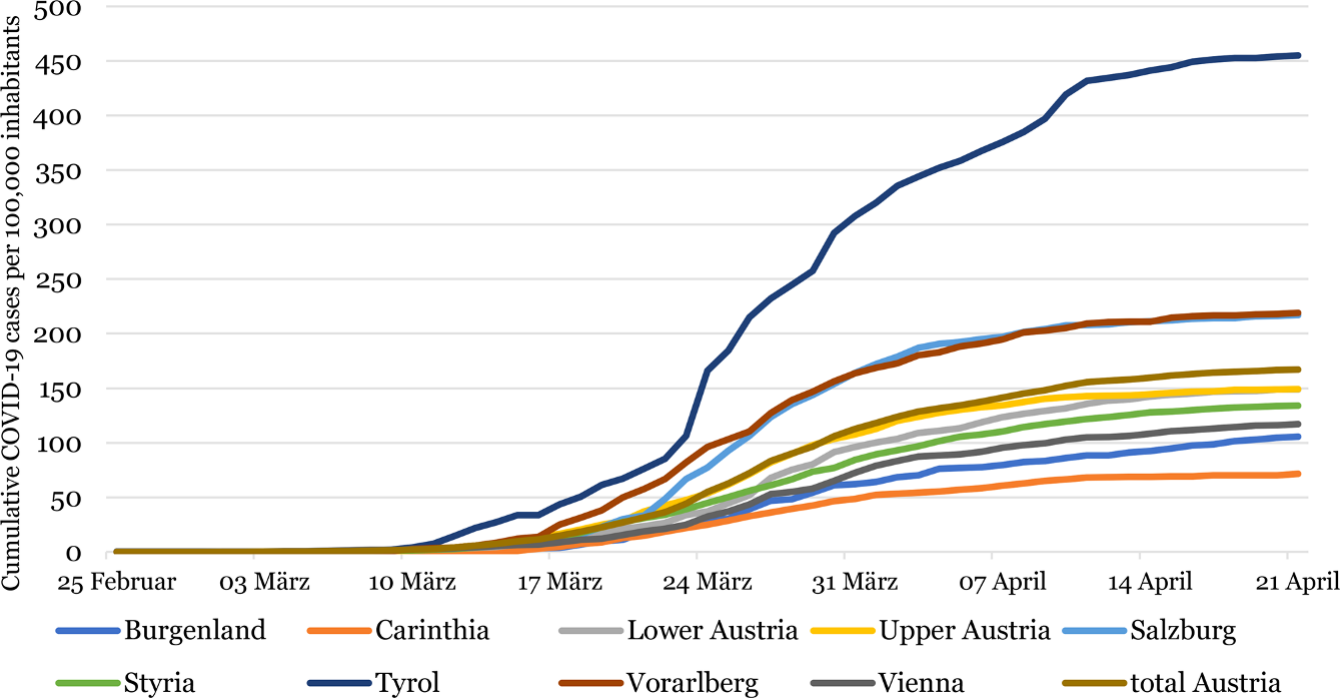
Cumulative COVID-19 cases per 100,000 inhabitants per Austrian province

Fig. 2 illustrates the substantial rise in the infection rate per 100,000 people, especially in Tyrol (one of the western Austrian provinces) which was one of the national epicentres and its subsequent halt by mid-April.

**Fig. 3:**
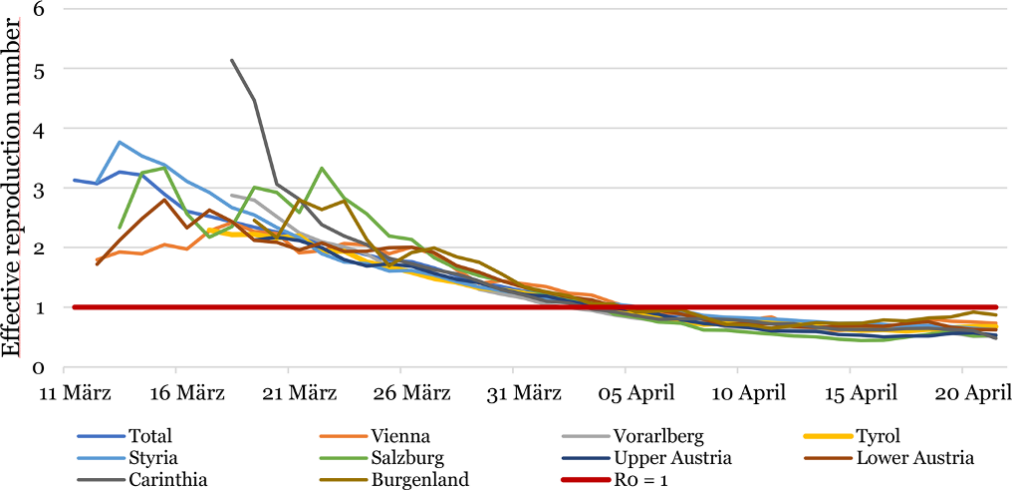
Effective reproduction numbers (R) per Austrian provinces

From the beginning of March to mid-March, the effective reproduction number (R), the net number of people to whom an infected person spreads the virus, was 1.81 in Austria. Later, between 20 March 2020 (4 days after the lockdown was imposed) and 1 April 2020, it decreased to 1.14, and finally it fell below 1 in the beginning of April. From 2 April 2020 to 20 April 2020 two calculations resulted in a stable number of 0.63 [19]. In Fig. 3, the originally heterogeneous effective reproduction numbers for the different provinces in Austria are depicted. The trend shows a gradual alignment and unitary dropping to R < 1 in the beginning of April.

#### RR of infection by sex

As of 20 April 2020, 51% of the total 14,810 cases were female, whereas the percentage of women in the total population was 50.8%, resulting in a RR of 1.01 (95% CI: 0.98–1.04; *p* = 0.63) for infection in women compared to men.

#### RR of death by sex

58% of the total 455 deaths from COVID-19 in Austria as of 20 April 2020 were attributable to men. With respect to the total population, this resulted in a significantly increased RR of death for men compared to women (RR = 1.44; 95% CI: 1.20–1.73; *p* < 0.0001). When assuming the cumulative infection numbers as population at risk, the RR of death for men compared to women somewhat diminished, but still differed significantly at 1.33 (95% CI: 1.11–1.61; *p* = 0.002).

#### RR of infection by age

An estimated 19% of the population was aged 65 years or older in the beginning of 2020. In the age group < 65 years, a total of 11,463 were tested positive for SARS-CoV‑2 and another 3347 infections occurred in the age group ≥ 65 years. According to these numbers, people aged 65 years and above had a 24% increased risk of being infected with SARS-CoV‑2 compared to the younger population (RR = 1.24; 95% CI: 1.20–1.29; *p* < 0.0001).

#### RR of death by age

An even stronger association was found when comparing risk of death in different age groups. Out of 455 deaths, 432 occurred in people aged ≥ 65 years leaving only 23 people under the age of 65 who died from the disease. This, with respect to the whole population, resulted in an 80-fold risk of dying from COVID-19 in people aged 65 years and over (RR = 80.07; 95% CI: 52.64–121.80; *p* < 0.0001) compared to the population < 65 years. Assuming again the cumulative cases to be the population at risk, RR for death for people aged ≥ 65 years was 64.33 (95% CI: 42.37–97.68; *p* < 0.0001) compared to the younger age group.

### Evidence on implemented public health measures

In the absence of any effective pharmacological public health measures such as an effective vaccine or an effective treatment, different strategies of nonpharmaceutical public health measures were adopted between February and April 2020 in Austria:

1. Avoidance of case importation,
2. Limitation of social contacts,
3. Hygiene measures,
4. Extensive testing,
5. Case tracking,
6. Call for public health research related to COVID-19.

#### Avoidance of case importation

The first approach was to limit the import of infections by imposing wide-ranging travel restrictions. First, passenger airplanes from risk regions, such as Italy, China, and Iran, were prohibited [20], health checks were conducted at random at the border to Italy [21], and a travel warning was issued, after which border control was implemented for passengers coming from Italy. Eventually, entry into Austria was prohibited for all passengers from Italy [22]. Other borders were subsequently closed as well and entry into Austria was only granted upon presenting a doctor’s letter and for Austrian citizens with obligation to self-quarantine for 14 days [23]. More than 7000 Austrian citizens were brought back from other countries via government initiatives [24]. Due to high case counts in Tyrol, at first certain municipalities [25] and later the whole province was put under quarantine to avoid spreading of infections [26]. Eventually some other municipalities across Austria were also put under quarantine during this 8‑week period [27]. This meant that only people with residency in Tyrol were allowed to enter the province [28] and anyone without such residency had to leave the province [29] and self-quarantine for 14 days thereafter.

#### Limitation of social contacts

Most implemented measures aimed at limiting the number of people gathering [30]. The idea of social distancing was communicated to the public: maintaining 1 m distance to others at all times as well as restricting social contacts to a minimum. One of the first measures taken was the prohibition of large public events of over 100 people indoors and over 500 people outdoors on 11 March [8]. In order to limit gatherings even further, universities and schools were closed and switched to home-learning mid-March [31, 32]. Furthermore, from 16 March a centralized lockdown, a general prohibition to enter public spaces [33], was imposed on all citizens. Entering public spaces and by extension leaving the house was generally forbidden unless it was within the scope of one of the four exceptions: going to work, shopping for essential goods, helping others and going outside for recreational walks. When outside, a distance of 1 m had to be maintained to all people not living in the same household [34]. The use of public transport was restricted to limited situations excluding the use for recreational activities [35]. Furthermore, nonessential shops (almost all except supermarkets, chemist’s shops and pharmacies) were forced to close and shortly thereafter bars and restaurants followed [34]. In further steps, federal gardens, playgrounds, sports facilities and sanatoriums were closed [36]. To avoid transmission at the workplace, employers were obligated to let people work from home whenever feasible [37]. By mid-April, small shops were allowed to reopen [38].

In order to prevent iatrogenic spread of the virus, doctor-patient contacts were reduced to a most essential minimum. In February people with respiratory symptoms were already asked to contact the health information hotline 1450 instead of going to a doctor’s office [39]. Later on doctor’s offices were requested to only receive emergencies and provide e‑prescriptions and medical certificates via telephone. Elective and preventive examinations, including some pregnancy check-ups, were postponed or cancelled as were elective surgeries in an attempt to spare intensive care capacity [40, 41]. Rehabilitation facilities were mostly closed [42] and in order to avoid importation of the virus into hospitals, visitors were largely prohibited [43].

#### Hygiene measures

General hygiene guidelines communicated by the government included [44]:

- Hand hygiene: frequent cleaning of hands with either soap or disinfectant
- Distance: keeping at least 1 m distance to anyone
- Face contamination: avoiding touching nose, eyes and mouth
- Respiratory etiquette: sneezing and coughing in tissue paper or elbow

**Fig. 4:**
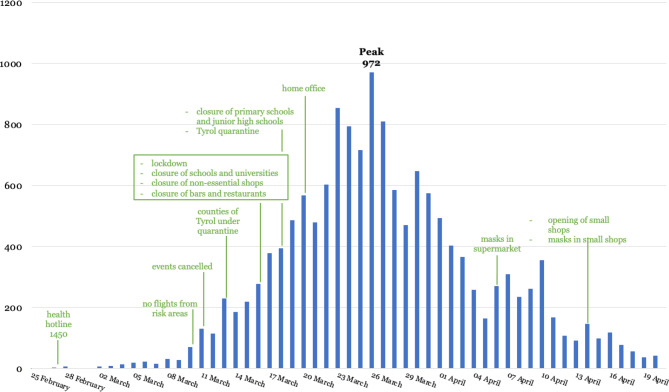
Number of new infections per day and timeline of main nonpharmaceutical public health measures in Austria

On 6 April the Austrian government as an additive measure imposed obligatory mask use in supermarkets [45]. Little over 1 week later, this was extended to public transport and small shops which were reopened at that time [38]. By 6 April a range of other hygiene requirements were put in place for supermarkets, such as provision of disinfectant, plexiglass shields at the checkout, regular disinfection of certain surfaces and limitations in the number of people allowed to enter the shop [46] (Fig. 4).

#### Extensive testing

Austria initially followed a testing strategy based on clinical symptoms [47]. In early stages, when testing capacities were still very limited, a combination of typical symptoms (fever, cough, etc.) and either contact to a confirmed case or stay in a high-risk area was defined as suspect case qualifying for a test [48]. Later on patients with symptoms without other explanation in need for hospitalization received testing as well [49, 50].

Additionally, there have been tests of asymptomatic essential workers (supermarkets, healthcare personnel) [51]. Furthermore, serial tests in retirement and nursing homes were being conducted [52]. At the end of April, a new testing strategy was announced which would focus strongly on the healthcare sector as well as on inhabitants of closed facilities and people who were transferred to these. Additionally, outpatient doctors would be given the authority to order a test without contacting 1450 in advance [53].

A study was commissioned by the government in an attempt to estimate the prevalence of active SARS-CoV‑2 infections and thereby the number of undetected cases in Austria. A representative sample of people were tested for active SARS-CoV‑2 infection at the beginning of April. The study resulted in an estimated prevalence of 0.33% active SARS-CoV‑2 infections in Austria (95% CI: 0.12–0.76%), translating into a total number of 28,500 actively infected people (confidence interval between 10,200 and 67,400 people) [54]. The official case counts of active infections during this time period ranged between 8325 and 8981 persons [4]. Two key messages could be drawn from this study: probable gross underestimation in official case counts and very low prevalence at sampling time. This low estimated prevalence corroborated the existing choice of testing strategies. The World Health Organization (WHO) advocated extensive testing for SARS-CoV‑2 but recommended selective testing in cases of limited resources prioritizing people at risk of developing severe disease and vulnerable populations, healthcare workers and symptomatic individuals in closed settings [55]. With an assumed prevalence as low as 0.33%, population-wide testing, even if technically feasible, was not deemed sensible as due to the low pre-test probability, the number of false positive results would likely be very high [56].

**Fig. 5:**
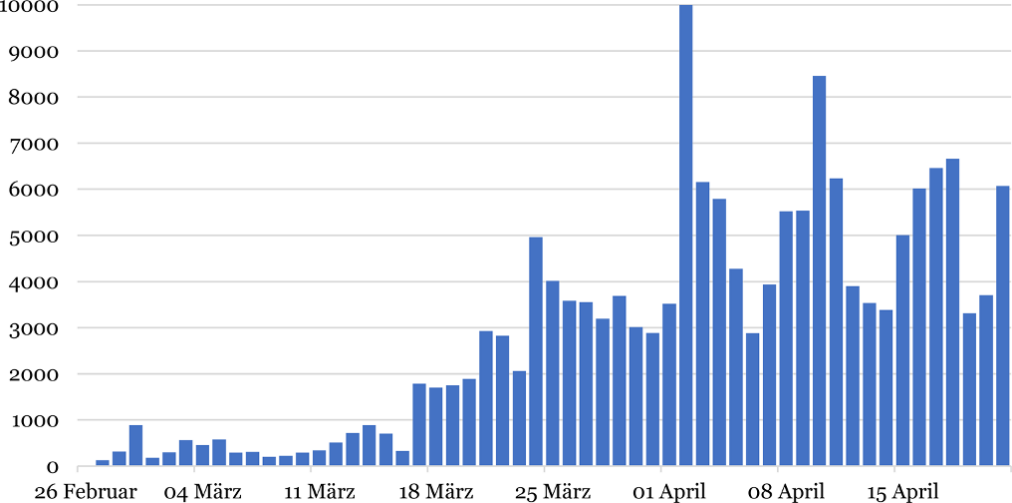
Number of polymerase chain reaction (PCR) tests for SARS-CoV‑2 carried out per day in Austria. Note: on 2 April, a high number of 36,327 tests were reported due to a lag in reporting which was not displayed in this graph

Fig. 5 shows the number of polymerase chain reaction (PCR) tests performed per day. The number of PCR tests for SARS-CoV‑2 conducted in Austria has increased substantially in the reporting period. First available data claimed 126 tests on 27 February 2020 and climbed up to a peak of 8456 tests on 10 April 2020 with considerable variations depending on the day. According to the ministry, depending on the availability of test reagents, Austria then had a capacity of up to 15,000 tests per day [52].

#### Case tracking

Another epidemiological approach implemented was contact tracing either through interviews [57] or through a smartphone application and was attempted with a voluntary Austrian version [58]. Acceptance of the application, however, was reported to be low [59].

#### Call for public health research related to COVID-19

Furthermore, several Austrian research funding agencies rapidly issued calls for COVID-19 related research. With a budget of 26 million Euros [60] (total funding budget of 833 million in 2018 [61]), the Austrian Research Promotion Agency (FFG) provided financial support for projects investigating basic research about the virus, infection prevention and control, development of therapeutics and diagnostics, planning and conduct of clinical trials as well as projects investigating new manufacturing strategies for medically critical areas such as protective gear [60]. The Austrian Science Fund (FWF) further supported research on humanitarian crises, such as pandemics using the example of SARS-CoV‑2 to gain knowledge for the management of future crises [62]. The Vienna Science and Technology Fund (WWTF) also issued a call for research [63].

### Acceptance of the implemented public health measures

In different waves of a public opinion survey starting 27–30 March and repeated 3 times during the first 8 weeks (3–8 April, 10–16 April, 17–21 April), the Austrian public opinion on the implemented public health measures was examined. The public in general had high acceptance rates, the highest level shown at the end of March when 71% of the respondents found the measures appropriate [64]. Throughout the four waves of surveys, the acceptance remained high with a slight increase in respondents deeming the measures “rather too extreme” or “too extreme” towards 21 April. Furthermore, over 60% of the respondents continuously rated the measures to be effective [64].

Another survey reported in the media found similar numbers with 73% of people thinking of the measures in general as appropriate at the end of March [65]. A similar proportion of respondents (74%) found that face mask wearing was justified in a survey conducted between 16 and 20 April [59].

### Evidence on the mental health impact of the pandemic

In the course of literature research, 15 articles were identified investigating the direct mental health impact of the COVID-19 pandemic and/or the consequently implemented public health measures which met all the inclusion criteria of our rapid review.

Out of the studies, 12 provided evidence for anxiety, 9 for depression, and 5 for PTSS. Two of the studies were found on the pre-print-server medRxiv and have not been peer reviewed to date. General characteristics of the studies included: all but three studies (Italy, Jordan, Iran) were conducted in China and all but one were online surveys. At the time of search, no studies had yet been available based on evidence from Austria. Demographics generally differed from average population in terms of sex ratio (more female), age distribution (younger) and education (higher levels of education).

#### Anxiety

Anxiety was named among the most common mental health impacts of COVID-19 [66]. In the identified studies, anxiety levels were measured using different instruments thus limiting the comparability of the measured anxiety levels. In general, prevalence rates in studied populations varied considerably from 6.3% to 50.9% [67–78]. The five studies using generalized anxiety disorder 7 (GAD7) scale showed considerably different prevalence rates between the studies. For instance, in an Italian study 20.8% of study participants reported severe anxiety symptoms [67], while in Jordan 13.1% were reported [70]. One study from China reported that 21.3%, 2.7% and 0.9% of the study participants had mild, moderate and severe anxiety levels, respectively [69]. Two other studies also from China reported moderate to severe anxiety symptoms in 22.6% [71] and 35.1% [68] of survey respondents.

The three studies using depression, anxiety and stress scale—21 items (DASS-21) to assess anxiety levels, showed similar results with 7.5% of survey respondents reporting mild anxiety, 20.4% moderate anxiety and 8.4% severe and extremely severe anxiety in China [72]. Interestingly, a follow-up study on the same population detected no significant changes in the mean stress, anxiety and depression scores over time, despite the substantial increase of confirmed COVID-19 cases between the first and the second wave of the survey [73]. An Iranian study reported mild, average, severe and very severe anxiety levels assessed with DASS-21 at 10.5%, 21.3%, 9.3% and 9.8% of the study respondents, respectively [74].

The studies using self-rating anxiety scale (SAS), observed lower prevalence rates of 6.3–9.6% for mild to severe anxiety in the studied populations [75–77]. A final survey carried out in China using the Beck-anxiety inventory reported increased anxiety levels in 29.0% (10.1% mild, 6.0% moderate, 12.9% severe) in the study cohort [78].

#### Depression

The levels of depressive symptoms ranged from 16.8% to 48.3% in the identified studies [67, 68, 70–73, 75, 77, 78]. In the Italian study depression was found at a rate of 17.3% among study respondents [67] and in Jordan 23.8% of respondents showed symptoms of severe depression [70].

#### Posttraumatic stress symptoms

Six papers investigating PTSS reported rates of elevated scores ranging from 4.6% to 53.8% [67, 72, 79–81].

#### Vulnerable groups

A number of the aforementioned studies also investigated differences in specific demographic groups and risk factors in order to identify those most at risk of mental health conditions who are possibly in need for intensified mental health attention during the pandemic. Studies showed that young age [67, 68, 74–78], being a student [72], better knowledge of the COVID-19 topics or more time spent on COVID-19-related topics [68, 75], and social media exposure [71] were associated with elevated anxiety levels. There was conflicting evidence towards sex groups. While some of the studies identified women to be more at risk for anxiety [67, 72, 74, 77], other studies claimed no difference [68, 69, 76, 78]. Risk factors for depression were similarly young age [67, 68, 75, 78] and being a student [72]. Concerning PTSS, being a student [72] and living in areas of high risk, i.e. Wuhan [79], and Northern Italy [67] were associated with higher levels of PTSS.

## Discussion

### Epidemiological indicators

In Austria, the number of new COVID-19 infections per day had peaked on 26 March, one month after the first confirmed case, and steadily decreased since, which is also reflected in the effective reproduction number dropping below one at the beginning of April. In the recent article about the time course of COVID-19 in Austria it was pointed out, however, that at the beginning of the Austrian epidemic many cases were imported and, hence, did not have infectious contacts in Austria which would result in an overestimation of R0 [82].

By 21 April, Austria had 14,810 confirmed cases, 455 deaths with a total number of about 189,000 tests having been conducted. The first German case was confirmed much earlier than in Austria, on 27 January [83]. With its approximately tenfold larger population, Germany reported about 143,000 cases by this day with about 4600 deaths and about 2 million tests done. These numbers are very much in line with the Austrian ones with a slightly higher level of testing. The first reported case of SARS-CoV‑2 in Italy was on 31 January. With its population of about 60 million, Italy has about 6.7 times the population of Austria. By April 21, it counted about 184,000 cases and a striking number of about 24,600 deaths, a much higher death toll than in Austria or Germany. By then 1.45 million tests had been carried out [84].

Case fatality ratio is a poor estimate during a pandemic, especially during its initial phase. There are many unknown variables, most importantly the number of total infections and total deaths. The common calculation of reported deaths divided by confirmed cases at that time lacks precision in many ways and it has shown to vary considerably with stages of the pandemic [85]. For comparison, using this simple calculation, by 21 April Germany had a case fatality ratio of 3.2, USA 5.4, China 5.4 and Italy as high as 13.3 [18]. A cross-temporal meta-analysis concluded a case-fatality ratio of COVID-19 in Europe of 4.0–4.5% [86, 87].

The increased RR of infection with SARS-CoV‑2 in older people has to be scrutinized as the Austrian testing strategies prioritized the risk group of older people and older people are more likely to suffer from severe diseases. Therefore, younger people who might be asymptomatic or only mildly affected might not have had the chance to be tested. The enormous risk increase for death in people aged ≥ 65 years, however, remains reason for concern.

### Public health measures

The Austrian public health interventions followed China’s example in many ways. China had established a wide variety of invasive measures amongst which were social distancing, home confinement, *cordons sanitaires*, centralized quarantine, traffic restriction and universal symptom survey. In China, these interventions presumably had a great effect of decreasing R from above 3.0 to below 1.0 in the beginning of February and decreasing it even further to below 0.3 in March [88].

In a compendium of systematic literature reviews conducted by the WHO in 2019 about nonpharmaceutical public health interventions in the context of influenza, there was no clear evidence in favor of any such measures except for case isolation, work closure, workplace measures and internal traffic restrictions [89]. The grade of evidence, however, was very low for these as well. There was moderate evidence on hand hygiene, where a meta-analysis could not show effectiveness against laboratory-confirmed influenza in community settings. The analysis, however, included studies with mixed findings [89]. Available evidence on the subject of face masks was also systematically investigated. A meta-analysis of ten randomized controlled trials did not find evidence that face masks were effective in reducing transmission of laboratory-confirmed influenza in a community setting, yet it was stated that there is mechanistic plausibility of effectiveness. There were no studies found on the effectiveness of respiratory etiquette. Although based on very low-grade evidence, other measures were also evaluated with the following conclusions: contact tracing (unknown), quarantine of exposed individuals (variable), school closures (variable), surface cleaning (lacking) and avoiding crowding (unknown). The following was stated about evidence on the effectiveness of travel restrictions: entry and exit screening (lacking), border closure (variable). In spite of the lack of evidence supporting these measures, a lot of them were still recommended in the context of influenza [89].

An interim report published by the WHO advocated wearing masks as a mitigation strategy for COVID-19 [90]. It was stated then that when worn by an infected person, spreading of infectious droplets can be prevented by a mask. They also reported limited evidence that a healthy individual can be protected in the household, when in contact with a sick person or when attending mass gatherings. It was, however, as mentioned above, also stated that at this point in time there was no evidence of the prevention of infections with SARS-CoV‑2 through universal community masking or masking in a wider community setting [90]. Officials, therefore, did not have the privilege of relying on solid scientific evidence when introducing nonpharmaceutical public health measures and had to act based on reports from other countries. In the meantime, a meta-analysis commissioned by the WHO has investigated several of the aforementioned measures and concluded considerable effectiveness in the reduction of SARS-CoV‑2 and COVID-19 transmission for physical distancing of 1m or more, and wearing face masks and eye protection [91].

Overall, in Austria, the rapidly decreasing number of daily new cases with a rising test capacity, suggests a strong effect of the public health measures taken. It is, however, still to be determined to what extent each of the measures contributed to the decrease in case counts so that politicians are able to make more informed choices about which measures to take in further stages of this or future epidemics. The communicated aim of flattening the curve so as to avoid overwhelming the healthcare sector had been more than achieved for the time being. The epidemic, however, is presumably far from over and it is unsure which public health measures will be needed to be taken until it is. The health effects of minimizing doctor-patient contacts during the lockdown and the resulting foregone care will be an important topic of future research in all areas of medicine.

### Mental health impacts

In the initial phase of the Austrian epidemic and the governmental measures little discussions arose about their direct mental health consequences. In our review we found that due to the wide extent of the COVID-19 pandemic, it may lead to a global mental health crisis and large-scale mental health interventions and mental healthcare disaster management plans may also have to be considered [66]. It was also shown that specific up to date and accurate health information and precautionary measures were associated with lower psychological impact and lower levels of anxiety and depression [72]. Our rapid review found no Austria-specific studies at that time.

By July 2020, a considerable number of studies on the mental health impacts of COVID-19 in Austria, however, have been published. For example, an online survey study by Pieh et al. [92], comparing the mental health impact in Austria to that in the United Kingdom reported moderate or severe anxiety levels in 19% of participants which is within the range reported by the early international studies 3.6%–35.1% [68, 69]. As for depression, the study found 21% of participants had moderate or severe depressive symptoms, while the Italian study using the same questionnaire found severe depressive symptoms in 17.3% of the respondents [67]. A study by Traunmüller et al. also reported high rates of psychological impact (moderate levels in 5.6% and severe levels in 37.7% of the participants) while severe depressive symptoms were present in 26.5% of participants, somewhat higher than those initially reported from China [72, 93]. Traunmüller et al. found significantly lower levels of anxiety and depression in men [93], while the international evidence was conflicting on that subject [67–69, 72, 74, 76–78]. They also found lower levels of education as well as unemployment to be significantly associated with higher levels of anxiety and depression [93]. Austrian studies are consistent with some international evidence on higher levels of anxiety and depression in younger age groups [67, 68, 75, 78, 93, 94]. Similar to the Chinese study [72], students in Austria were identified to be more vulnerable to suffer from depression during the pandemic [94]. Due to the large number of ongoing studies and expected to be published in the near future on the subject, a separate systematic review of all Austrian studies on the mental health effects of COVID-19 is likely to be imminent.

## Conclusion

To conclude, this is a rapidly evolving situation and probably the best documented public health emergency in history. It will, therefore, be subject to important future research not only on the direct consequences on people’s health, lives and well-being, but also on the long-term health, healthcare and socioeconomic impacts.
